# European Central Bank׳s monetary policy decisions: A dataset of two decades of press conferences

**DOI:** 10.1016/j.dib.2018.08.061

**Published:** 2018-08-28

**Authors:** Thierry Warin, William Sanger

**Affiliations:** a HEC Montréal, Canada; b Polytechnique Montréal, Canada; c CIRANO, Canada

## Abstract

The dataset gathers all press conferences in text form made by the first three Presidents of the European Central Bank since its inception in 1998. Press conferences are composed of two elements: (1) introductory statements and (2) questions and answers (Q&A) from journalists. They serve as the main communication vehicle about the monetary policy decision of the ECB׳s Governing Council. From 1998 to 2016, a total of 205 press conferences have been delivered. The dataset is structured into two main sections: (1) 205 introductory statement of the Presidents of the ECB explaining the monetary policy of the Institution and (2) 205 answers provided by the Presidents to the journalists, hence a total of 410 statements (914,499 words or 3050 pages) about the monetary policy and its context, in text form.

## Specifications Table

**Table:** 

Subject area	*Economics*
More specific subject area	*Monetary Policy, Institutional Communication*
Type of data	*Text corpus, Figures, Excel-file*
How data was acquired	*Data extracted from the Institution׳s portal*
Data format	*Raw, filtered*
Experimental factors	*For the second part of the dataset (Q&As), the data concern the answers provided by the ECB*
Experimental features	*Texts were structured using the R programming language. This database could be used to understand the perception of institution through its communication.*
Data accessibility	*The Data is made available in the supplementary material coming with this article.*

## Value of the data

• The dataset offers a unique access to almost 20 years of institutional communication.
• The dataset can be used at once to analyze the speeches of the introductory statements of the ECB as well as the answers provided by the Presidents to the journalists during the Q&A segment.
• The dataset can be used to analyze structured as well as unstructured data. The dataset has been precoded: on top of a corpus of text machine-readable (CSV file), the number of words, and questions asked by intervention have been recorded.

## 1 Data

The dataset provided with this article is a CSV file gathering all Introductory Speeches of the European Central Bank. Throughout the database, a total of 205 statements are provided in raw format. Moreover, after detailing the monetary policy of his institution, the President of the European Central Bank traditionally reply to the journalists covering the Introductory Speeches. The answers to those questions are provided in the CSV file. The next table describes the different variables of the data file shared with this article.

Description of the variables of the dataset:

**Table:** 

**Name of the variable**	**Type**	**Description**
date	date	date of the Speech
president	{1; 2; 3}	name of the President (1: W. Duisenberg; 2: J.-C. Trichet; 3: M. Draghi)
firstPart	text	Introductory Speech
firstPartVp	{1; 0}	1: the Vice-President intervened during the first part; 0 otherwise
secondPart	text	Answers to the journalists
secondPartExternalIntervention	{1; 0}	1: someone else than the President intervened during the second part; 0 otherwise
vicePresident	{1; 0}	1: the Vice-President intervened during the second part; 0 otherwise
numberOfAnswers	number	number of answers provided by the President during the Q&A section
link	text	link to the original document (redirecting on the ECB׳s online portal)

To the best of our knowledge, this is the first dataset allowing for a comprehensive analysis of the relevant communications for the ECB monetary policy [1]. This paper has also some academic and managerial implications [2], [3]. As such, through the use of unstructured data (Presidents’ speeches), researchers can provide a quantitative perspective on the communications generated by the ECB regarding the Eurozone. In term of managerial implications, the same methodologies could be applied to CEO communications or firms annual reports and their implications in terms of perceptions from the financial markets.

This dataset gathers a corpus of introductory statements from October, 13th of 1998 to December, 8th of 2016. In total, 205 press conferences are detailed. Moreover, the answers provided by the three Presidents complete this dataset. In addition to the text, a first layer of coding has been provided for both parts of the statements(the number of words). For the second part of the statements, we manually counted the number of answers provided to the journalists.

## 2 Experimental design, materials and methods

Data was manually acquired through the Institution׳s online portal.^1^ The dataset is presented as tidy data [4], i.e. each line is an observation, each column is a variable and each element of the dataset is a value (either text, dummy variable, date…).

The number of words could be assessed using the following R code of the stringr library in R [5]:

(1) speeches$nbOfWordsFirstPart<−str_count(speeches$firstPart,"//S+")

**Fig. 1:**
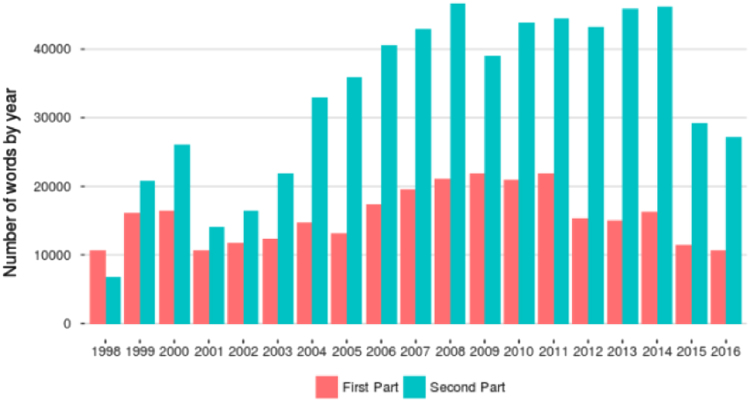
Number of words by year for each part of the Introductory Statements of the ECB (1998–2016).

In Fig. 1, we provide the total number of words by year for each part of the dataset.

**Fig. 2:**
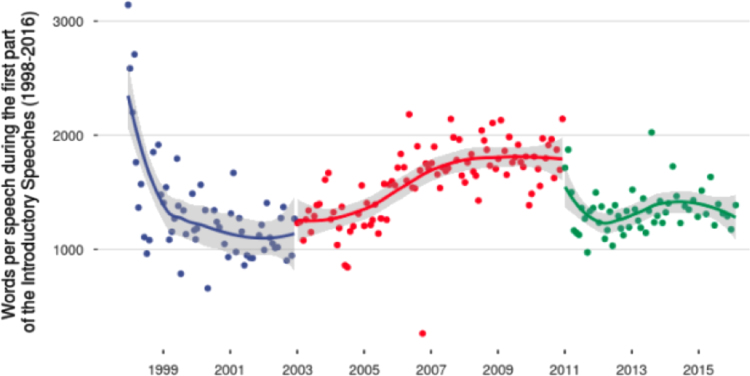
Number of words by each President of the ECB during the first part of the press conferences (blue: Wim Duisenberg; red: Jean-Claude Trichet; green: Mario Draghi).

Fig. 2 breaks down the number of words for each President during the first part of the Introductory Speeches.

**Fig. 3:**
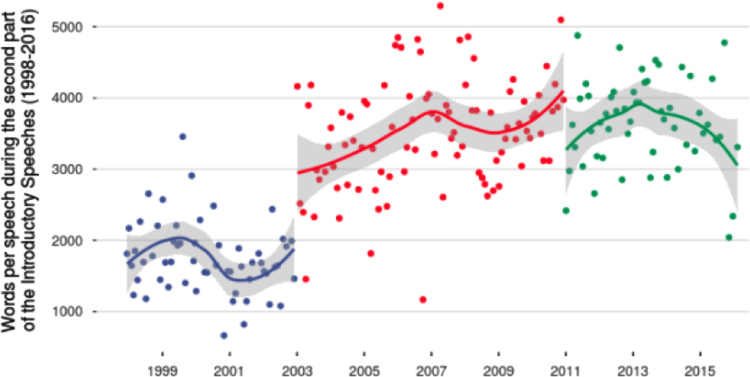
Number of words by each President of the ECB during the second part of the press conferences (blue: Wim Duisenberg; red: Jean-Claude Trichet; green: Mario Draghi).

Finally, Fig. 3 provides an overview of the number of words for the Q&A part of the press conferences by each President. From this database, quantitative methodologies of text processing could be applied. As such, this database could be extended and complement research on Central Bank communications, especially in the Eurozone, as well as financial markets’ perceptions.

## Footnotes

1 https://www.ecb.europa.eu/press/pressconf/.
